# Chest radiotherapy after left upper lobectomy may be a risk factor for thrombosis in the pulmonary vein stump

**DOI:** 10.1186/s13019-022-01902-x

**Published:** 2022-06-13

**Authors:** Cheng-Yang Song, Daisuke Kimura, Ikuo Fukuda, Fumiyasu Tsushima, Takehiro Sakai, Takao Tsushima

**Affiliations:** 1 Department of Thoracic and Cardiovascular Surgery, Hirosaki University Graduate School of Medicine, 5 Zaifu-cho, Hirosaki, Aomori 036-8562 Japan; 2 Department of Diagnostic Radiology, Hirosaki University Graduate School of Medicine, Hirosaki, Aomori Japan; 3 Department of Thoracic Surgery, Fourth Affiliated Hospital of China Medical University, 4 Chongshan Road, Shenyang, 110032 China; 4 Department of Cardiovascular Surgery, Suita Tokushukai Hospital, Senriokanishi, Suita-shi, Osaka-fu 565-0814 Japan; 5 Department of Internal Medicine, Tokiwakai Hospital, Sakaki, Minamitsugarugun Fujisakimachi, Aomori 038-1216 Japan; 6 Department of Thoracic Surgery, Hirosaki Hospital, National Hospital Organization, Tominocho, Hirosaki, Aomori 036-8174 Japan

**Keywords:** Left upper lobectomy, Pulmonary vein stump thrombus, Postoperative chest radiotherapy

## Abstract

**Background:**

Thrombosis in the pulmonary vein stump (PVS) is not a well-known complication after pulmonary lobectomy, but it has the potential to cause embolism to vital organs. The aim of this study was to evaluate the risk factors for thrombosis in the PVS after pulmonary lobectomy.

**Methods:**

A total of 439 patients who underwent pulmonary lobectomy from 2008 to 2017 were retrospectively reviewed, and 412 patients were further analyzed. The state of the PVS was evaluated by chest contrast-enhanced computed tomography (CECT). Univariate analysis was performed to evaluate the potential risk factors for thrombosis in the PVS.

**Results:**

Thrombosis in the PVS was detected in 6 of 412 (1.5%) patients, and 5 of them underwent left upper lobectomy (LUL) (5/100, 5.0%) (*P* = 0.004). In the analyses of the LUL group, postoperative chest radiotherapy was identified as a risk factor for thrombosis in the PVS (*P* = 0.024), and postoperative atrial fibrillation showed a tendency to be a risk factor for thrombosis (*P* = 0.058).

**Conclusions:**

Chest radiotherapy after LUL is a possible risk factor for thrombosis in the PVS. Periodic chest CECT is recommended after postoperative chest radiotherapy for patients after LUL.

## Background

Thrombosis in the pulmonary vein stump (PVS) is not a well-known complication after pulmonary lobectomy, but it has the potential to cause embolism to vital organs. Left upper lobectomy (LUL) has been considered to have a higher risk of thrombus formation in the PVS than other lobectomies [1–3]. Previous studies showed that the length of the left superior pulmonary vein (LSPV) stump was significantly longer than that of other PVSs due to the anatomical aspect of the resected pulmonary vein [1, 4]. The long LSPV stump resulted in stasis of blood flow and subsequent thrombus formation [2, 3]. Although LUL is considered to be a risk factor for thrombus formation in the PVS, relevant research reports are still rare, and the risk factors for thrombosis in the LSPV stump remain unclear. In the present study, the risk factors for thrombus formation in the PVS after pulmonary lobectomy were evaluated, with special focus on the risk factors in the LUL group.

## Methods

### Selection of patients and management of clinical data

A total of 439 patients who underwent pulmonary lobectomy in Hirosaki University Hospital from January 2008 to December 2017 were retrospectively reviewed. Patients who underwent partial resection, segmentectomy, multiple lobectomy, and pneumonectomy were not included, and 27 patients who did not undergo chest contrast-enhanced computed tomography (CECT) at least once within 2 years after surgery were excluded. Thus, 412 patients remained for the further analyses.

### Evaluation of thrombosis in the PVS

Chest CECT images were retrospectively interpreted to check for PVS thrombus by three doctors, including two thoracic and cardiovascular surgeons and one radiologist.

### Operative policy

Anatomical lobectomy with systematic regional lymph node dissection was performed for primary lung cancer (*N* = 396). The extent of the lymph node dissection was determined according to the criteria of the Japan Lung Cancer Society [5]. Anatomical lobectomy was performed for metastatic carcinoma of the lung (*N* = 14) and for benign disease (*N* = 2) in the present study. The pulmonary vein was dissected in the extrapericardial space by ligation or linear stapler as a routine procedure.

### Statistical analysis

Statistical analyses were carried out using the Statistical Package for the Social Sciences (SPSS) (version 25, IBM, Armonk, NY, USA). Univariate analysis was used to predict the risk factors for thrombosis in the PVS. A significant difference was accepted as a *P* value less than 0.05 for all analyses.

## Results

### Patients’ characteristics

A total of 412 patients (261 males, 151 females) were analyzed in the present study. The patients’ ages ranged from 22 to 84 years (median 68 years). The operative procedures consisted of 100 left upper lobectomies (LULs), 73 left lower lobectomies (LLLs), 146 right upper lobectomies (RULs), 22 right middle lobectomies (RMLs), and 71 right lower lobectomies (RLLs). The operative approaches were 318 video-assisted thoracic surgery (VATS) procedures and 94 open thoracotomies. The tumor pathologies included 394 cases of primary lung cancer, 14 cases of metastatic lung tumors, 2 cases of pulmonary sarcoma, and 2 cases of benign tumor. Overall, 138 cases received postoperative chemotherapy, and 61 received postoperative chest radiotherapy.

### Thrombosis in the PVS after pulmonary lobectomy

**Fig. 1:**
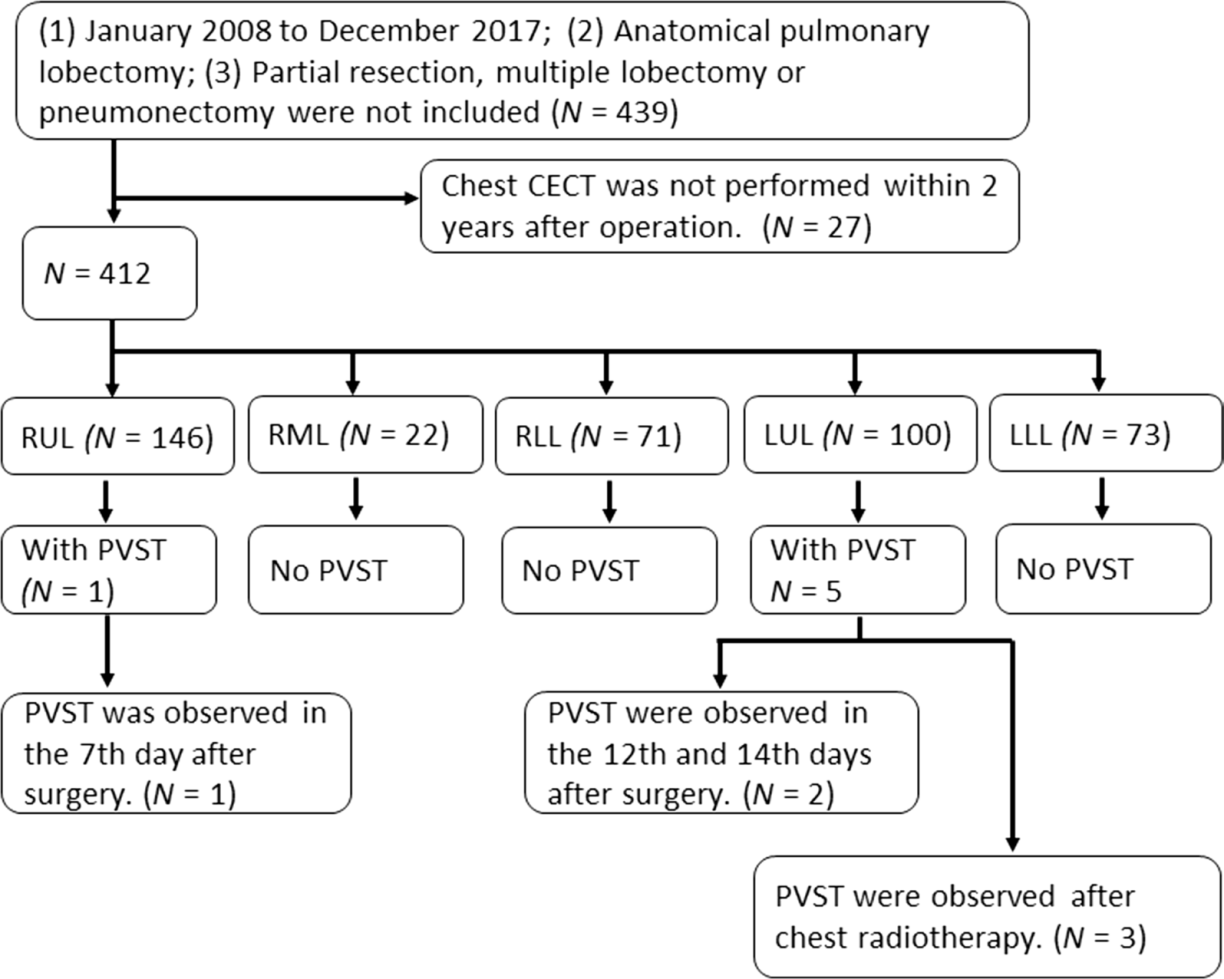
The flow chart of the study. *CECT* chest contrast-enhanced computed tomography, *LUL* left upper lobectomy, *LLL* left lower lobectomy, *RUL* right upper lobectomy, *RML* right middle lobectomy, *RLL* right lower lobectomy, *PVST* pulmonary vein stump thrombus

**Fig. 2:**
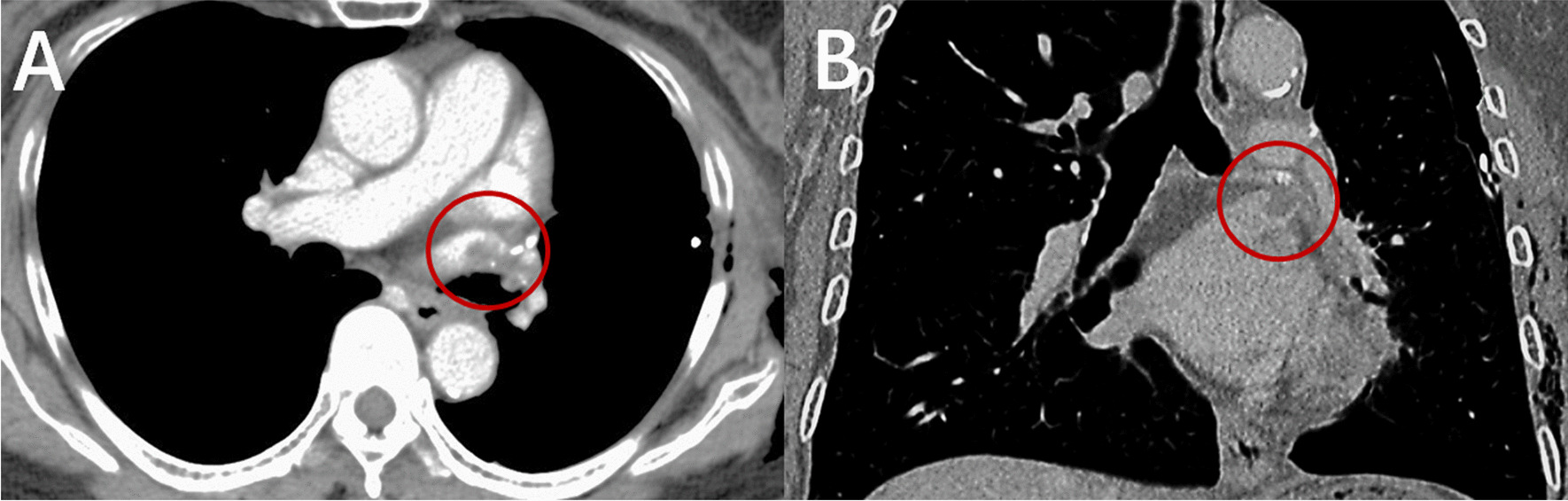
Typical radiological findings of thrombosis in the pulmonary vein stump after pulmonary lobectomy.** a** Thrombosis in the left superior pulmonary vein stump was detected by postoperative chest contrast-enhanced axial computed tomography scans;** b** Thrombosis in the left superior pulmonary vein stump was detected by postoperative chest contrast-enhanced coronal computed tomography scans

Thrombosis in the PVS was observed in 6/412 (1.5%) patients after pulmonary lobectomy, including 5 patients after LUL and 1 patient after RUL (Table 1, *P* = 0.004). Figure 1 shows the flow chart. Figure 2 presents the typical radiological findings of thrombosis in the PVS after pulmonary lobectomy.

**Table 1:** Univariate analyses of operative procedures associated with PVS thrombosis

	Patients with thrombosis (n = 6)	Patients without thrombosis (n = 406)	*P* value
Procedure (1), n (%)			
LUL	5 (83)	95 (23)	0.019
LLL	0 (0)	73 (18)	
RUL	1 (17)	145 (36)	
RML	0 (0)	22 (5)	
RLL	0 (0)	71 (18)	
Procedure (2), n (%)			
LUL	5 (83)	95 (23)	0.004
Non-LUL	1 (17)	311 (77)	

*PVS* pulmonary vein stump, *LUL* left upper lobectomy, *LLL* left lower lobectomy, *RUL* right upper lobectomy, *RML* right middle lobectomy, *RLL* right lower lobectomy

### Risk factors for thrombosis in the PVS after LUL

In the patients who underwent LUL, clinicopathological factors were compared between the patients with and without PVS thrombus. Table 2 shows the result of the univariable analysis to evaluate the possible risk factors associated with PVS thrombus formation after LUL. It was found that postoperative chest radiotherapy was significantly related to thrombus formation in the PVS (*P* = 0.024). Postoperative atrial fibrillation (AF) showed a tendency to be a risk factor for thrombosis (*P* = 0.058).

**Table 2:** Univariate analyses to identify the risk factors for thrombosis in the LSPV stump

Variable	Patients with thrombosis (n = 5)	Patients without thrombosis (n = 95)	*P* value
Age, years, mean value (range)	69 (63–75)	67 (39–80)	0.557
Sex, n (%)			
Male /female	4 (80)/(20)	54 (57)/41 (43)	0.329
Smoking index, mean value (range)	853 (0–1290)	574 (0–2250)	0.313
CEA, ng/mL, mean value (range)	3.9 (2.1–7.4)	7.3 (0.5–152)	0.658
Comorbidity, n (%)			
Hypertension	1 (20)	38 (40)	0.389
Diabetes mellitus	1 (20)	19 (20)	1.000
Hyperlipidemia	0 (0)	9 (10)	0.999
Preoperative chemotherapy, n (%)	0 (0)	3 (3)	0.999
Preoperative chest radiotherapy, n (%)	0 (0)	0 (0)	N/A
VATS, n (%)	4 (80)	69 (73)	0.719
PVS treatment, n (%)			
Ligation/stapler	0 (0)/5 (100)	7 (7)/88 (93)	0.999
Together /respective	3 (60)/2 (40)	80 (84)/15 (16)	0.184
Operative time, minutes, median value (range)	290 (228–399)	249 (157–530)	0.175
Blood loss, ml, mean value (range)	255 (58–590)	223 (20–2900)	0.858
Atrial fibrillation, n (%)	2 (40)	9 (10)	0.058
Chemotherapy, n (%)	1 (20)	29 (31)	0.621
Chest radiotherapy, n (%)	3 (60)	14 (15)	0.024

Smoking index: the average root number per day multiplied by smoking years of smoking

*LSPV* left superior pulmonary vein, *CEA* carcinoembryonic antigen, *VATS* video-assisted thoracic surgery, *PVS* pulmonary vein stump

### Main details of the cases with thrombosis in the LSPV

**Fig. 3:**
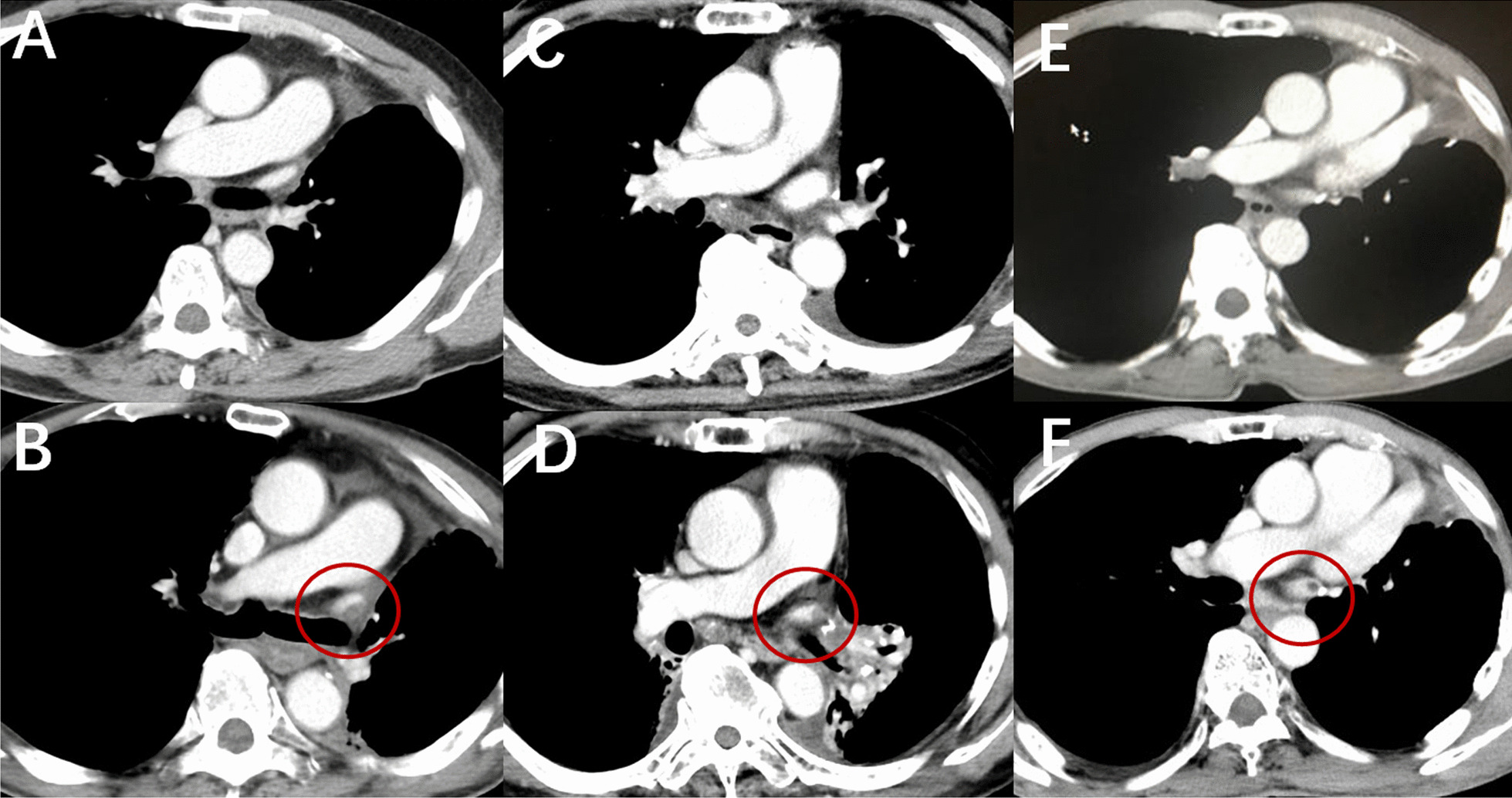
Typical radiological findings of thrombosis in the pulmonary vein stump after postoperative chest radiotherapy. **A**, **C**, **E** No thrombus is seen after left upper lobectomy before chest radiotherapy; **B**, **D**, **F** Thrombosis is seen in the pulmonary vein stump after postoperative chest radiotherapy

The 5 cases with thrombosis in the PVS after LUL are shown in Table 3. Thrombosis was detected on the first postoperative chest CECT in case 1 and case 2 at 12 days and 14 days after surgery, respectively. Thrombosis was not detected on the first postoperative chest CECT in cases 3, 4, and 5, but it was detected on chest CECT after postoperative chest radiotherapy (Fig. 3). Case 3 and case 5 received 60-Gy radiotherapy to the mediastinal region, and case 4 received 45-Gy radiotherapy to the right lung field. Postoperative AF was detected in case 4 and case 5. Case 1 and case 4 received anti-coagulant drug therapy, and the thrombus disappeared after treatment. However, the 3 other cases did not receive antithrombotic therapy; thrombus disappeared spontaneously in 2 cases and became smaller in 1 case. Acute renal infarction was detected in case 1 in the 12 days after surgery. The other 4 cases did not develop acute organ infarction.

**Table 3:** Clinical characteristics of patients with thrombosis in the PVS after LUL

Case number	Sex	Age, (years)	Interval^a^ (days)	Interval^b^ (days)	Detected by the first postoperative chest CECT	Postoperative chest RT	Postoperative AF	Follow-up of thrombus
Case 1	M	70	12	N/A	Yes	No	No	Anti-coagulate drug/ disappeared
Case 2	F	72	14	N/A	Yes	No	No	No treatment/disappeared
Case 3	M	64	365	310	No	Mediastinal RT (60 Gy)	No	No treatment/became smaller
Case 4	M	75	976	154	No	Right lung RT (45 Gy)	Yes	Anti-coagulate drug/disappeared
Case 5	M	63	1127	688	No	Mediastinal RT (60 Gy)	Yes	No treatment/disappeared

*PVS* pulmonary vein stump, *LUL* left upper lobectomy, *Gy* gray, *CECT* chest contrast-enhanced computed tomography, *RT* radiotherapy, *AF* atrial fibrillation

^a^Time from left upper lobectomy to the first detection of thrombosis in the PVS

^b^Time from the start of postoperative chest radiotherapy to the first detection of thrombosis in the PVS

## Discussion

In this study, the risk factors for thrombosis in the PVS after lobectomy were investigated. It was confirmed that LUL was a risk factor for thrombosis in the PVS after lobectomy. More importantly, postoperative chest radiotherapy was found to be significantly associated with thrombosis in the LSPV stump in patients who underwent LUL.

Some researchers have reported LUL as a risk factor for thrombosis in the PVS [1–3]. The mechanism of the thrombosis in the LSPV stump was considered to be related to two main factors. One was the hemodynamic changes (stasis or turbulence) caused by a long LSPV stump [1, 2, 6], and the other was vascular endothelial injury of the PVS caused by surgery [6, 7]. Ohtaka [2] considered that thrombus was more likely to develop within a few days after LUL, meaning that short-term thrombosis was closely related to the operation.

**Fig. 4:**
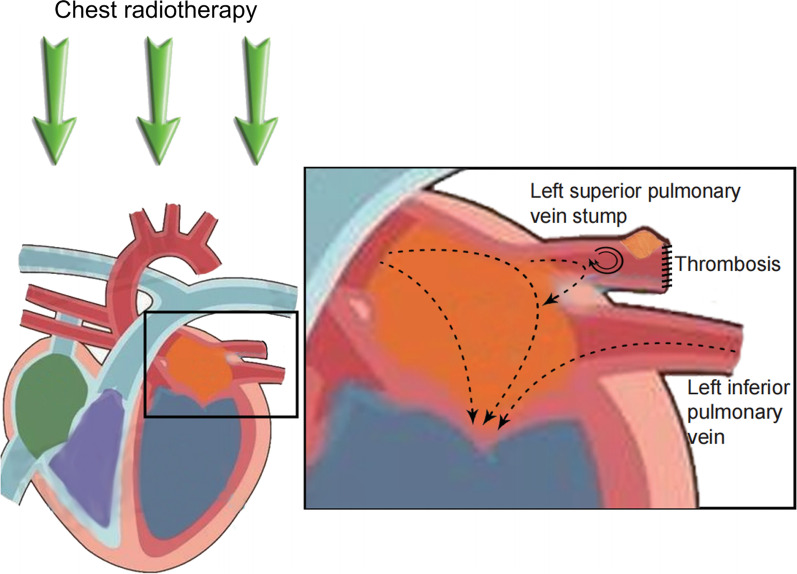
Pattern diagram of thrombosis in the left superior pulmonary vein stump after chest radiotherapy

In the present study, there were 3 cases with thrombosis in the PVS within 2 weeks of surgery, including 2 cases after LUL and 1 case after RUL. However, 3 cases with thrombosis in the PVS were observed 365–1127 days after LUL, and the PVS thrombus was not observed on the first postoperative chest CECT. All 3 cases with thrombosis in the LSPV stump received postoperative radiotherapy. We considered that the thrombus that developed a long time after the operation, especially thrombus that was not detected on the first chest CECT after surgery, might not be closely related to the operation. Radiotherapy was considered to be a risk factor for endothelial vascular injury [8–10]. Some researchers suggested that high-dose radiation to the chest wall may cause intimal injury, leading to endothelial disruption and activation of myofibroblasts and platelets. Endothelial injury results in the formation of cholesterol plaques containing infiltrates of macrophages and neutrophils, which have been associated with plaque hemorrhage and an increased risk of coronary thrombosis [11–13]. Chest radiotherapy might aggravate the injury of the vascular endothelium in the PVS, which might be an important reason for thrombus formation in the PVS. We consider that postoperative chest radiotherapy is a risk factor for thrombosis in the PVS. The pattern diagram was provided in Fig. 4.

Atrial fibrillation (AF) was considered to increase the risk of vital organ infarction by the formation of a thrombus in the left atrial appendage due to turbulent blood flow [14, 15]. In the present study, AF was found to be a potential risk factor for thrombus formation in the LSPV stump. AF might not only result in turbulent blood flow in the left atrial appendage, but it may also aggravate blood stasis in the LSPV stump after LUL, which might be the reason for the high risk with AF for thrombosis in the LSPV stump. However, this hypothesis needs further experimental verification.

Thrombus in the PVS was found to disappear spontaneously without any anticoagulant therapy (Table 3), which was also reported by Hattori et al. [16]. Therefore, some developed thrombus might have disappeared when the patient underwent chest CECT. We considered that the timing of chest CECT had an effect on the detection of thrombosis in the PVS. More thrombosis in the PVS might be observed if chest CECT is routinely performed shortly (within 2 weeks) after pulmonary lobectomy.

## Conclusion

LUL was a risk factor for thrombosis in the PVS. Postoperative chest radiotherapy was a risk factor for thrombosis in the LSPV stump after LUL. Periodic chest CECT is recommended after postoperative chest radiotherapy for patients who underwent LUL.

### Limitations

This study has several limitations: (I) potential selection bias, given the nature of a retrospective study; (II) potential bias caused by the nonuniform chest CECT time; and (III) potential bias caused by the limited number of cases.

## Data Availability

Not applicable.

## Abbreviations

PVS: Pulmonary vein stump
LUL: Left upper lobectomy
LLL: Left lower lobectomy
RUL: Right upper lobectomy
RML: Right middle lobectomy
RLL: Right lower lobectomy
LSPV: Left superior pulmonary vein
SPSS: Statistical Package for the Social Sciences
VATS: Video-assisted thoracic surgery
AF: Atrial fibrillation
CECT: Contrast-enhanced computed tomography
